# The Role of Defendant Gender and PTSD Diagnosis in a Battered Spouse Case

**DOI:** 10.1177/08862605241257594

**Published:** 2024-06-22

**Authors:** Rebekah Adair-Russell, Krystia Reed, Maria F. Torres

**Affiliations:** 1 The University of Texas at El Paso, USA

**Keywords:** battered spouse syndrome, PTSD, mental illness, intimate partner violence

## Abstract

A quarter of women and 11% of men report being survivors of intimate partner violence (IPV) during their lifetimes in the United States. Despite being victims themselves, people who kill their IPV abuser can still be subject to criminal proceedings. Given this complexity, the law has employed battered spouse syndrome (BSS) as a tool used in some jurisdictions to support a claim that an IPV survivor killed in self-defense. A defendant who is attempting to claim self-defense using BSS may introduce testimony of post-traumatic stress disorder (PTSD). However, a diagnosis of PTSD can pose problems in admission during litigation as the occurrence of a traumatic event is often what is being decided. The present study examined how college students, living on the U.S.-México border, perceive survivors-turned-defendants in a BSS mock trial. Specifically, we had each participant read a written trial transcript of a mock trial where gender of the defendant and clinical diagnosis of PTSD were manipulated. The current study hypothesized that jurors would be more lenient toward female defendants than male defendants (*Hypothesis 1*), jurors would be influenced by a PTSD diagnosis of the defendant (*Hypothesis 2a–b*), and female jurors would be more lenient than male jurors (*Hypothesis 3*). We also wanted to examine the impact of victim blaming, sexism, stigma of PTSD, and prior exposure to IPV on decision-making (*Hypothesis 4a–d)*. Findings showed jurors were more lenient with female defendants than male defendants, however there was no effect of clinical diagnosis except on difficulty of decision. Implications of the role defendant gender has in decision-making is discussed.

A recent viral defamation case involving Johnny Depp and Amber Heard had many people glued to their screens. The case initially began when Heard published a story in 2018, on the heels of the *#MeToo* movement, describing her survival of intimate partner violence (IPV) (Rosenblatt, 2022). Although Heard did not name her abuser, people speculated it was Depp. Later, Depp sued Heard for defamation, claiming that the accusations she made in her article were false. The defamation case resulted in extensive testimony about abuse suffered by both Heard and Depp. Despite the general perception that only women experience IPV, ultimately, this case highlighted that men can also be IPV survivors (National Coalition Against Domestic Violence [NCADV], 2021).

The Johnny Depp and Amber Heard situation is unfortunately not unique. In the United States, approximately 20 people per minute are victims of IPV (NCADV, 2021), which includes any sexual violence, physical violence, and/or stalking by an intimate partner (Moorer, 2021). In fact, estimates suggest one in four women and one in nine men are victims of IPV in their lifetime (Truman & Morgan, 2014). However, these numbers may be underestimated because most IPV incidents go unreported (Conwill, 2010). Moreover, IPV incidents are often dangerous, with 33% resulting in serious injury to the victim (NCADV, 2021). Because IPV survivors are in constant danger and fear, some jurisdictions within the legal system have developed the concept of battered spouse syndrome (BSS) as a justification in court for self-defense when the IPV survivor kills their abuser.

## Battered Spouse Syndrome

BSS is a term used to explain the experiences of female abuse survivors to explain why people may remain in an abusive relationship (Flynn, 2019). The typical cycle of violence involves (a) tension building, (b) an abusive incident, (c) reconciliation, and (d) a “honeymoon” phase, which is relatively peaceful until the next tension building (L. E. Walker, 1979). Given that the cycle may result in extreme violence at any point, the survivor often lives in constant fear (Fair, 2018), resulting in some survivors feeling helpless and as if their only option is to kill their abuser (L. E. Walker, 1992).

If the survivor is charged with killing their abuser, they may want to bring in testimony about BSS to help overcome societal assumptions that a person can leave an abusive relationship. Usually, if the killing occurs during an abuse event in which the survivor is in imminent danger, the survivor can claim self-defense to protect themselves from criminal liability (Emerson, 1996). All fifty states allow for expert testimony explaining BSS when the survivor is claiming self-defense (U.S. Department of Justice & U.S. Department of Health and Human Services, 1996).

On the other hand, some survivors kill their abuser when they are not in imminent danger, thus eliminating claims of self-defense as an option. Only 20% of states permit BSS testimony if the defendant first presents evidence that they have been battered, regardless of whether or not claiming self-defense is an option (U.S. Department of Justice & U.S. Department of Health and Human Services, 1996). Legal cases involving IPV and BSS can be particularly complex, and jurors are often required to make difficult decisions about responsibility. Psychological research suggests that many factors may influence juror decisions, including gender bias, juror gender, and stigma.

### Gender Bias

Initially, it was assumed that only women experienced BSS. In fact, it was originally termed Battered *Woman* Syndrome (L. E. Walker, 1979). However, this conceptualization has been criticized for being based on gender and racial stereotypes which suggest it only applies to White, cisgender, heterosexual, female survivors (Crenshaw, 2012; Sokoloff & Dupont, 2005). Thus, the language has changed to BSS in order to be more inclusive by also recognizing experiences of men and non-binary individuals (Holliday et al., 2022).^1^ Yet, as demonstrated by the initial media reports in the Heard-Depp case, public perception has not necessarily kept up with research. Two types of gender bias have the potential to impact juror decisions in murder cases where BSS testimony is offered: defendant gender and juror gender.

#### Defendant Gender

Women are less likely to be involved in the justice system than men (Bontrager et al., 2013), partially because men commit more crimes than women (J. T. Walker & Maddan, 2013). However, evidence suggests that differences are partially due to gender bias entering the decision-making process when the decisionmaker has discretion, resulting in leniency toward female defendants. In the past, police officers used their discretion and were less likely to arrest a woman for committing the same crime as a man (Krohn et al., 1983). Yet, more recently, IPV risk assessment tools have shown to help mitigate police discretion and are rated by the police as useful resources when assessing IPV calls (Campbell et al., 2018). Prosecutors can also use their discretion and are less likely to charge a woman for the same crime as a man (Romain & Freiburger, 2013). Juries use their discretion and are less likely to convict a woman (Livingston et al., 2019). And, if they are arrested, charged, and convicted, judges tend to use their discretion to give women shorter sentences with lesser likelihood of incarceration than men who were convicted of the same crime (Freiburger & Sheeran, 2020). Eliminating discretion in the decision-making process can reduce gender differences. For example, reducing judicial discretion by setting mandatory minimum sentences has reduced gender discrepancies in sentencing for drug offenses (Gaskins, 2004).

Initial explanations for gendered leniency in legal decisions tended to rely on the chivalry hypothesis (Curran, 1983). The chivalry hypothesis posits that men (such as police or judges, two primarily male professions) view women as less dangerous, culpable, and threatening and, therefore, are more lenient than they are with male defendants (Freiburger, 2010; Gruhl et al., 1984). The chivalry hypothesis is declining in popularity, especially as support for gender equality increases (Bontrager et al., 2013). Moreover, there are also instances where women are treated more harshly than men. For example, women with a past criminal history received harsher sentences than men (Doerner & Demuth, 2014). Thus, the role of gender in legal decision-making is complicated, and a closer examination of gender and the role it can play in less “traditional” trial scenarios (e.g., when a male is claiming BSS) is important to explore.

##### Defendant Gender and BSS

Research suggests people have a hard time recognizing men as survivors of crime (Donnelly & Jenyon, 1996; Haegerich & Bottoms, 2000), which might be particularly problematic for men trying to use BSS as a defense. If a man is not seen as a victim of BSS, then he will likely be unsuccessful in using it as a defense. Gender norms may be to blame for gender bias in jurors’ decisions, particularly when discussing self-defense. Typically, men are physically larger and stronger, and thus, it is difficult for people to imagine men as survivors of abuse from a woman (Migliaccio, 2002; Hodell et al., 2014). Consistent with these gender stereotypes, male abuse survivors report being met with minimization of their experience and ridicule when seeking support services (A. Walker et al., 2020).

In fact, one study directly relevant to the current study investigated the role of abuser gender and height in juror decisions during a BSS murder trial involving a heterosexual couple (Hodell et al., 2014). Jurors were more lenient with women, who were less likely to be found guilty, and were shown more sympathy than men who killed their abusive partners. However, there was an interaction between height and gender. This effect was unexpected, especially considering female defendants are often looked at more favorably than male defendants (Livingston et al., 2019), so it is unclear why shorter females would be punished more.

However, support for egalitarianism is on the rise, with 96% of Americans believing men and women should have equal rights (Horowritz, et al., 2017). Thus, it is possible that attitudes about male abusers have changed in the past 10 years since the Hodell et al. (2014) article. Moreover, Hodell et al. (2014) did not examine the influence that juror gender had on decision-making in their study. Therefore, it is necessary to see if these findings replicate in 2023, as well as examine the role of juror gender.

#### Juror Gender

Juror gender might also play a role in legal decision-making. In general, female jurors are often more lenient in their verdicts than male jurors (Bell et al., 2021; Mossière et al., 2018). But, in cases involving sexual violence (e.g., sexual assault and rape), female jurors are more likely to convict the defendant, regardless of gender, than male jurors (Pozzulo et al., 2010; Schutte & Hosch, 1997). Meaux et al. (2018) found that in assault cases, regardless of survivor gender, female jurors were more confident in their guilty verdicts of female defendants. Other studies have shown that female jurors are more likely to find female defendants not guilty (Cutroni & Anderson, 2021; Hodell et al., 2014).

However, BSS cases provide a unique situation—the abuse survivor becomes the accused defendant. Women are typically more educated about and familiar with IPV than men (Greene et al., 1989; Woodzicka & LaFrance, 2001). Thus, female jurors might be more understanding and lenient toward the defendant, an IPV survivor who killed their partner, regardless of the gender of the defendant. Some research has shown that men are more likely to find the “victim-turned-defendant” guilty in BSS cases (Eigenberg & Policastro, 2016).

##### Sexism and Victim Blaming

Sexism is another factor that has been found to influence verdicts in BSS cases (Cutroni & Anderson, 2021). Specifically, researchers have found that people who score higher in hostile sexism, which involves negative views toward women who violate traditional gender roles (Glick & Fiske, 2001), are more likely to find the victim-turned-defendant guilty in a intimate partner homicide case (Cutroni & Anderson, 2021). Men who subscribe to hostile sexism beliefs justify abuse through the idea that it is their job to keep women in line (J. T. Wood, 2004). On the other hand, benevolent sexism which involves perceiving women as weak and in need of help (Glick & Fiske, 2001) has not been found to influence decisions in BSS cases (Cutroni & Anderson, 2021). Benevolent sexism beliefs approach justification of abuse through victim blaming (Masser et al., 2010; for a more thorough review see Connor et al., 2017). However, more research is needed to examine whether ambivalent sexism influences perceptions of cases involving men on trial for killing their female abuser.

### Clinical Diagnosis

Another factor that might influence decisions in a BSS case is whether the defendant has a clinical diagnosis. BSS itself is not a clinical diagnosis in the Diagnositc and Statistical Manual of Mental Disorders (DSM-V; American Psychiatric Association, 2013). However, many of the symptoms are covered by post-traumatic stress disorder (PTSD), which involves exposure to an event with a threat of violence, serious injury, or death and is recognized in the DSM-V (American Psychiatric Association [APA], 2022; Flynn, 2019). Common symptoms of PTSD include distressing memories of the traumatic event(s), nightmares, being easily startled or frightened, negative thoughts about self, hopelessness, memory problems, and detachment from friends/family (APA, 2022). Thus, these symptoms are consistent with the cyclical pattern of IPV abuse (tension, abuse, and “honeymoon”/reconciliation phases), resulting in common BSS symptoms: hyperarousal, re-experiencing the trauma/battering, and intimacy issues (L. E. Walker, 1979, 2006). In fact, women who have survived IPV show high rates of PTSD because of the abuse (estimates range from 31% to 97%; Golding 1999; Khadra, 2015; Kemp et al., 1995).

A defendant who is attempting to claim self-defense using BSS may attempt to show they have a PTSD diagnosis as evidence that the abuse occurred (Holliday et al., 2022). Yet, PTSD testimony can pose problems in self-defense murder cases as the occurrence of a traumatic event is often what is being litigated. Whether or not a PTSD diagnosis is admissible depends on the jurisdiction. Some jurisdictions consider PTSD irrelevant and inadmissible (Berger et al., 2012) as the case itself is to determine if the defendant was using self-defense during a supposed traumatic event. However, in other jurisdictions, a defendant may be permitted to bring in such a diagnosis through the testimony of an expert witness (Berger et al., 2012). Although the standard to determine whether expert testimony is permissible varies based on jurisdiction, PTSD diagnoses have been permitted under the three most common standards (*Daubert*, *Frye*, and Federal Rules of Evidence; Berger et al., 2012).

On the one hand, it is possible that a clinical diagnosis helps defendants (e.g., Mossiere & Maeder, 2016; L. Wood et al., 2014). However, there are also concerns that bringing in a diagnosis might backfire and create prejudice against the defendant. Research suggests there is significant stigma against people with mental illness, resulting in perceptions that the individual is violent or not credible (Curcio & Corboy, 2020; Levi, 2022). However, limited research has examined PTSD—the one vignette study directly examining PTSD found that a diagnosis of PTSD resulted in increased public stigma (Thibodeau & Merges, 2024). Moreover, research has not examined the role of a PTSD diagnosis in a BSS case specifically.

### The Present Study

The current project examined how gender of the defendant, juror gender, and PTSD diagnosis impact juror decision-making in a criminal case involving a BSS defense. We have several hypotheses (*Hypotheses 3–4d*) that have been pre-registered with the Open Science Foundation (https://osf.io/3c2ne/?view_only=76376ef42bef4e129b6785dee31158e2).

Based on prior research, we hypothesized that jurors will be more lenient on female defendants than male defendants (*Hypothesis 1*). Given the mixed research on mental illness in the courtroom, we have competing hypotheses: Jurors will be more lenient on a defendant with a PTSD diagnosis because there is more evidence of BSS (*Hypothesis 2a*), or jurors will be less lenient on a defendant with a PTSD diagnosis because of stigma (*Hypothesis 2b*). We expected that female jurors would be more lenient than male jurors because the case involves IPV (*Hypothesis 3*). We did not expect any interactions between these variables.

We also had several hypotheses regarding covariates examining individual juror differences. We hypothesized that jurors who score higher on victim blaming (*Hypothesis 4a*), hostile (but not benevolent) sexism (*Hypothesis 4b*), and PTSD stigma (*Hypothesis 4c*) would be more likely to find the defendant guilty, but those with greater exposure to IPV would be less likely to find the defendant guilty (*Hypothesis 4d*).

## Method

All materials are available to view on the Open Science Framework (https://osf.io/3c2ne/?view_only=76376ef42bef4e129b6785dee31158e2) and were approved by the Institutional Review Board at The University of Texas at El Paso.

### Participants

We conducted a power analysis using the “pwr” package in R which indicated we needed a minimum of 180 participants to detect an effect size of 0.25 with 80% power and 4 conditions (Champely, 2020). We ran the project for the entire semester and ended up recruiting 421 participants. We removed 33 participants for failing attention checks, a further 90 participants for failing manipulation checks, and 62 participants for not completing an entire scale. Thus, our final sample consisted of 236 undergraduate students recruited from the university participant pool, with 230 used for analysis due to missing values on certain measures. Participants were compensated with partial course credit for completion.

Most of the participants were female (69.5%), identified as Hispanic (86.9%), and had an average age of 21 (*SD* = 3.347). The most frequently cited political party affiliations were Democrat (39.8%) followed by “not political” (33.9%); most people either leaned liberally (44%) or in the middle (39.8%) of political views.

### Measures

#### Trial Transcript

Participants read a 25-page transcript of a criminal trial involving second-degree murder in which the defendant claimed that they had previously been abused by the survivor and, therefore, wanted to invoke the BSS defense (based on Lavalle v. Regina, 1990). The transcript varied in two ways: the defendant’s gender and PTSD diagnosis. All couples were heterosexual, so the gender manipulation either described a female defendant/IPV survivor with a deceased male partner/accused abuser or a male defendant/IPV survivor with a deceased female partner/accused abuser.

Each party called two witnesses. The prosecution first called an eyewitness, John Evans, who testified that he was at a party at the house shared by the defendant and deceased. He noticed them arguing and going upstairs, then he heard gunshots and found the defendant deceased in the bedroom. Next, the prosecution called police officer Steve Russell who first responded to the scene. He testified that the defendant admitted to shooting the deceased. Then, the defense called the defendant, who testified their partner had repeatedly abused them. On the day of the incident, the defendant said their partner had been verbally abusive (e.g., calling the defendant worthless), had engaged in acts of physical aggression (e.g., throwing a vase toward the defendant), and had threatened to kill the defendant after the party. Finally, Jane Roll, a clinical psychologist served as an expert witness for the defense. She testified about her mental health evaluation of the defendant and explained that the deceased had abused the defendant. Depending on condition, the expert also testified that she clinically diagnosed the defendant as having PTSD. The trial transcript concluded with closing statements from the attorneys and judicial instructions.

#### Attention and Manipulation Check

To ensure participants were paying attention, they were asked three attention check questions throughout the survey. Participants were excluded if they failed two attention check questions. After reading the transcript, participants were asked three additional manipulation check questions (gender of the defendant, gender of the decedent, and whether there was a PTSD diagnosis). Participants were excluded if they failed one manipulation check question.

#### Verdict

##### Binary Verdict

Participants were asked to determine the verdict of the defendant in a trichotomous option of guilty, not guilty, or not guilty by reason of self-defense. Close to 30% indicated the defendant as guilty, 2.1% selected not guilty, and 68.2% determined the defendant was not guilty by reason of self-defense. Due to the limited number of pure “not guilty” verdict responses (*n* = 5), the final verdict variable was dichotomized as “guilty” or “not guilty” (which included both not guilty and not guilty by self-defense) for analyses.

##### Scaled Verdict

Participants also rated their confidence in their verdict from 0 (not at all) to 100 (very). Overall, respondents indicated participants were confident in their verdict decision (*M* = 74.87, *SD* = 18.17). This was then recoded into a scaled verdict variable by making the scores of the people who voted not guilty negative. Thus, scores ranged from −100 (very confident in not guilty verdict) to 100 (completely confident in guilty verdict).

##### Difficulty

Participants also rated difficulty reaching their verdict from 0 (not at all) to 100 (very). Therefore, smaller values indicate less difficulty in making decisions compared to higher numbers indicating more difficulty (*M* = 54.96, *SD* = 29.33).

#### Covariates

We assessed several individual difference variables as covariates in this study (see Table 1 for statistics for each scale). First, we assessed victim blaming using the Victim-Blaming Attitudes–Intimate Partner Violence Against Women (VB-IPVAW) Scale, a 12-item measure that assesses the extent to which people blame victims for IPV (Martín-Fernández et al., 2018). Next, we assessed sexism using the Ambivalent Sexism Inventory (ASI), a 22-item measure assessing the extent to which people agree with sexist beliefs (Glick & Fiske, 1996). The scale is divided into two subscales: benevolent sexism which measures traditional views of women (e.g., “Men should sacrifice to provide for women”) and hostile sexism which measures prejudice against women (e.g., “Most women interpret innocent remarks as sexist”). Third, we measured PTSD stigma using the Mental Illness Stigma Scale (MISS, Day et al., 2007) which we reduced from 28-items to 16 relevant items and modified to focus specifically on PTSD rather than general mental illness. Our measure focused on PTSD stigma in the five sub-categories of treatability, relationship disruption, anxiety, visibility, and recovery. Finally, we asked about IPV exposure. Our two-item measure is separated between experience in their personal life and exposure in the media.

**Table 1.** Descriptives for Covariates.

Measure	*M*	*SD*	Likert Scale Range	Current α	Original α
Victim blaming (VB-IPVAW)	17.30	6.24	1 (low victim blaming) to 5 (high victim blaming)	.86	.89
Sexism: Benevolent (ASI)	1.33	0.85	1 (low benevolent sexism) to 5 (high benevolent sexism)	.71	.83
Sexism: Hostile (ASI)	2.19	0.82	1 (low hostile sexism) to 5 (high hostile sexism)	.85	.92
PTSD stigma (modified MISS)	59.07	10.51	1 (low PTSD stigma) to 7 (high PTSD stigma)	.66	.71
IPV exposure: Personal	.98	1.16	0 (never) to 4 (very frequently)	—	—
IPV exposure: Media	2.43	1.07	0 (never) to 4 (very frequently)	—	—

*Note.* Prior α values come from the original scale validation papers. VB-IPVAW = Victim-Blaming Attitudes–Intimate Partner Violence Against Women Scale; ASI = Ambivalent Sexism Inventory; MISS = Mental Illness Stigma Scale.

### Design and Procedure

The present study was a 2 (defendant gender: male vs. female) × 2 (clinical diagnosis: none vs. PTSD) between-subjects design. After providing informed consent, participants were randomly assigned to condition. They read the trial transcript, which varied based on defendant gender and whether the expert provided a clinical diagnosis. Participants then rendered a verdict, completed the individual difference measures, and provided demographic information.

## Results

### Binary Verdict

In order to assess the role of defendant gender, clinical diagnosis, and juror gender on verdict, we first conducted a binary logistic regression (see Table 2 for full logistic regression model). The dichotomous verdict (guilty vs. not guilty/not guilty by reason of self-defense; reference group: not guilty).

**Table 2.** Binary Logistic Regression Examining Binary Verdict Decision.

Variables	*B*	*SE*	Wald $\chi^{2}$	Odds ratio	95% CI [*LL*, *UL*]
Defendant Gender	−0.927	0.313	8.779*	0.392	[0.214, 0.731]
Defendant PTSD	0.336	0.310	1.175	1.399	[0.762, 2.567]
Participant Gender	−0.009	0.362	.001	0.991	[0.487, 2.017]
Victim Blaming	−0.036	0.030	1.395	0.965	[0.910, 1.024
Hostile Sexism	0.488	0.234	4.361*	1.629	[1.030, 2.575]
Benevolent Sexism	−0.462	0.223	4.314*	0.630	[0.407, 0.974]
Mental Health Stigma	−0.017	0.016	1.050	0.983	[0.952, 1.015]
Familiarity in Contact	0.179	0.146	1.511	1.196	[0.899, 1.591]
Familiarity in Media	−0.121	0.162	.558	0.886	[0.646, 1.217]
Constant	1.457	1.093	1.778	4.292	

*Note.* Overall model χ^2^(9, *N* = 230) = 19.27, *p* = .023, −2 log likelihood = 256.46, Cox and Snell *R*^2^ = .08, Nagelkerke *R*^2^ = .12.

* *p* < .05.

**Figure 1.**
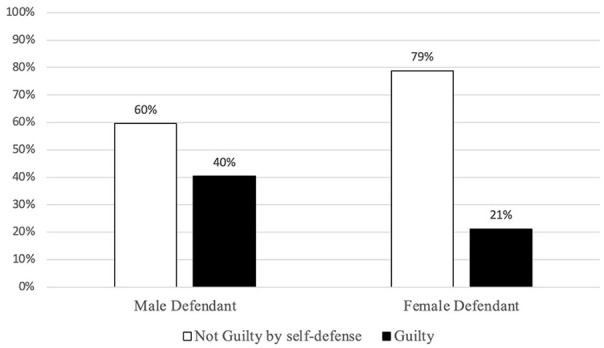
Frequencies of binary verdict on defendant gender.

Consistent with predictions (*Hypothesis 1*), female defendants were significantly less likely to be found guilty than male defendants (see Figure 1). Contrary to expectations, neither PTSD diagnosis (*Hypothesis 2*) nor juror gender (*Hypothesis 3*) significantly predicted the verdict.

Our predictions regarding individual differences *(Hypothesis 4*) were partially supported. As can be seen in Table 2, consistent with *Hypothesis 4b*, participants who scored higher on hostile sexism were *more* likely to find the defendant guilty. On the other hand, although we did not expect an effect of benevolent sexism based on prior research, participants who scored higher on benevolent sexism were *less* likely to find the defendant guilty. Victim blaming (*Hypothesis 4a*), mental illness stigma (*Hypothesis 4c*), and prior exposure to IPV (*Hypothesis 4d*) were not significant predictors of verdict.

### Scaled Verdict

In order to examine a wider range of verdict perceptions, we conducted an Analysis of Covariance (ANCOVA) using the scaled confidence in verdict as the dependent variable, defendant gender, clinical diagnosis, and juror gender as independent variables, and the covariates examining individual difference measures (victim blaming, sexism, mental illness stigma, and prior exposure to IPV; see Table 4 in Supplemental Materials for full model statistics). Higher scores on scaled verdict indicated greater confidence in a guilty verdict, whereas lower scores indicated greater confidence in a not guilty verdict.

As expected (*Hypothesis 1*), there was a main effect of defendant gender, *F*(1, 216) = 6.06, *p* = .015, $\eta_{\text{p}}^{2}$ = .03. Participants were more confident that female defendants were not guilty (*M* *=* −45.26, 95% CI [−56.98, −33.54], *SE* = 5.95) than male defendants (*M* *=* −18.93, [−32.14, −5.73], *SE* = 6.70). However, once again, contrary to expectations neither clinical diagnosis (*Hypothesis 2a–b; F*(1, 216) = 1.46, *p* = .23, $\eta_{\text{p}}^{2}$ = .00) nor participant gender (*Hypothesis 3; F*(1, 216) = .04, *p* = .84, $\eta_{\text{p}}^{2}$ = .01) significantly changed scaled verdict.

**Figure 2.**
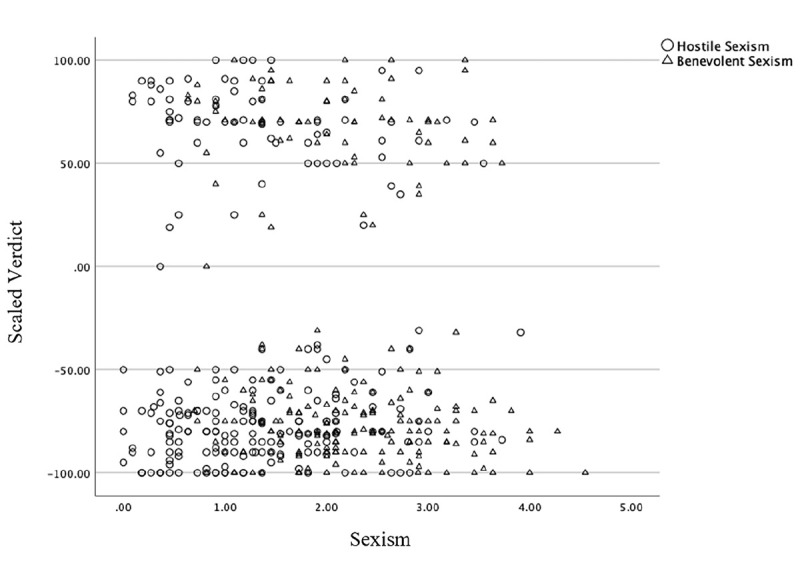
Scatterplot of sexism on scaled verdict.

In terms of individual differences (*Hypothesis 4*), only sexism (*Hypothesis 4b*) was a significant covariate. In the ANCOVA, both hostile (*F*[1, 216] = 5.11, *p* = .03, $\eta_{\text{p}}^{2}$ = .02) and benevolent sexism (*F*[1, 216] = 6.35, *p* = .01, $\eta_{\text{p}}^{2}$ = .02) were significant covariates (see Figure 2). A follow-up correlation indicated that only benevolent sexism significantly correlated with scaled verdict, *r* = −.16, *p* = .014. As benevolent sexism increased, people were less confident in their guilty verdicts. However, hostile sexism did not significantly correlate with scaled verdict, *r* = .02, *p* > .05. Contrary to expectations, victim blaming (*Hypothesis 4a; F*[1, 216] = 1.60, *p* = .21, $\eta_{\text{p}}^{2}$ = .01), PTSD stigma (*Hypothesis 4c; F*[1, 216] = 1.38, *p* = .24, $\eta_{\text{p}}^{2}$ = .01), and prior IPV exposure (*Hypothesis 4d; F*_personal_[1, 216] = 1.71, *p* = .19, $\eta_{\text{p}}^{2}$ = .01; *F*_media_[1, 216] = 0.11, *p* = .74, $\eta_{\text{p}}^{2}$ = .00) were not significant covariates and did not correlate with scaled verdict.

### Difficulty

Finally, to examine whether defendant gender, clinical diagnosis, and participant gender influenced how difficult the decision was, we ran the same ANCOVA as for scaled verdict but used the continuous measure of difficulty as the outcome variable. Contrary to our hypotheses, defendant gender (*Hypothesis 1*) was not significantly related to perceptions of difficulty.

As expected, clinical diagnosis (*Hypothesis 2a*) was a significant predictor of difficulty, *F*(1, 216) = 5.75, *p* = .02, $\eta_{\text{p}}^{2}$ = .03, where those with a PTSD diagnosis were rated as more difficult cases (*M* = 60.76, *SD* = 3.30).

Contrary to our hypotheses, participant gender (*Hypothesis 3*), nor any individual differences (*Hypothesis 4*), victim blaming (*Hypothesis 4a*), sexism (*Hypothesis 4b*), stigma (*Hypothesis 4c*), and familiarity with IPV (*Hypothesis 4d*) were not significant predictors of difficulty.

An unexpected three-way interaction was found among defendant gender, defendant PTSD, and participant gender, *F*(1, 216) = 4.98, *p* = .03, $\eta_{\text{p}}^{2}$ = .02 (see Table 5 in Supplemental Materials for full report). Post hoc analyses showed that among men the condition of the trial with the male defendant who had a PTSD diagnosis was a more difficult decision (*M* = 24.83, *SD* = 11.81).

## Discussion

The present study examined how defendant gender, clinical diagnosis, and juror gender influenced juror perceptions in a BSS case. We had two primary outcome variables: verdict (both binary and scaled) and difficulty reaching the decision. Although we had similar hypotheses for both outcomes, our results diverged.

In terms of defendant gender, we found the predicted main effect on verdict (both binary and scaled; *Hypothesis 1*): Female defendants were treated more leniently in this study than male defendants. Thus, we replicated prior findings of gendered leniency (Livingston et al., 2019), even in a violent murder case. Given that we held the case details consistent in all conditions, these results suggest that under the same circumstances, female defendants might receive more leniency than male defendants. There was no influence of the defendant gender on perceptions of difficulty.

Although this leniency in verdict occurred after controlling for sexism, it is worth noting that both hostile and benevolent sexism significantly predicted both verdict variables. Increased hostile sexism was related to greater perceptions of defendant guilt; increased benevolent sexism was related to lesser perceptions of defendant guilt. In fact, these results appear to be consistent with the chivalry hypothesis, in which people who believe women need to be saved are more likely to be lenient with them (Curran, 1983) despite this hypothesis currently being out of favor. Moreover, these results support scholarly recommendations for assessing sexism beliefs in *voir dire* may help mitigate gender bias in juror decisions (Becker & Swim, 2012; Cutroni & Anderson, 2021).

On the other hand, when it came to clinical PTSD diagnosis, the pattern was flipped: Clinical diagnosis did not significantly influence either type of verdict but was related to perceptions of difficulty. However, this was not quite what was predicted in *Hypothesis 2a* or *2b*. Instead, we found that cases where a PTSD diagnosis was present resulted in participants finding it more difficult to make their decisions. Yet the clinical diagnosis did not have any impact on verdict or confidence in verdict. Together, these findings suggest that jurors are considering the PTSD diagnosis, but it is not powerful enough to impact the ultimate verdict.

Finally, we examined juror gender and covariates exploring individual differences (sexism, victim blaming, mental illness stigma, or exposure to IPV). There were significant findings involving sexism, discussed above; however, the additional individual differences did not have a significant influence on juror decisions. Given prior research that female jurors are more understanding of sexual assault survivors than male jurors (Cutroni & Anderson, 2021; Hodell et al., 2014), it was particularly surprising that juror gender did not influence verdict. Perhaps this finding was due to the sample largely consisting of women.

### Future Directions and Limitations

One of the strengths and weaknesses of this study was the sample. On the one hand, psychology research has been criticized for using primarily WEIRD samples that lack diversity. The present sample was mostly Latinx-identifying students located in the U.S.-México border region. This population has not been widely researched, particularly on this topic, making the Latinx perspective a strength of this study. The lack of diversity of race/ethnicity in psycholegal research is problematic because all races and ethnicities are eligible for jury duty; therefore, it is our responsibility to account for diversity in legal research. Therefore, finding support for several of our hypotheses in a primarily Latinx sample provides support for generalizability of the findings. However, this study was not specifically designed to examine differences in decision-making based on participant race or ethnicity. Future research might consider a more direct examination to better understand generalizability of these findings and others.

On the other hand, the sample comprised college students, most of whom were women. Research comparing college students and community members indicates that for most jury decisions, there is no difference, and there is no reason we should expect it in this study (Bornstein et al., 2017), particularly because the university has more non-traditional college students than many schools. However, the same study indicates we might have concerns that women and men would make different decisions in this case, given it involves sexual assault. Most of the studies relied upon in the meta-analysis are very dated, though. Given the #MeToo movement and other societal changes emphasizing the prevalence of sexual assault, more research should be conducted to examine current gender differences in these types of trials to fully understand the extent to which a sample with mostly women can generalize more broadly.

Similarly, future studies would benefit by examining non-heterosexual relationships. Although this study was able to offer a new perspective by varying the gender of the accused abuser, all of the couples were heterosexual. In the future, research should examine perceptions of same-sex couples, particularly in relation to existing gendered stereotypes.

Finally, using a more robust methodology where jurors can see the parties (e.g., pictures or videos) would be beneficial to future research. We limited our materials to a written transcript in order to offer experimental control. Other research has shown that attractive defendants receive more lenient sentences than unattractive defendants (Darby & Jeffers, 1988; LaFrance & Hecht, 1995). Attractiveness of both parties might be a particularly interesting variable to examine in these types of cases.

## Conclusions

Given that research suggests both women and men can be victims of IPV, it is important to consider whether these victims-turned-defendants are treated equally in the legal system. Moreover, it is important to understand whether a clinical diagnosis is helpful to a defendant by providing evidentiary support or harmful by triggering bias. The current study examined these important questions by manipulating defendant’s gender and clinical PTSD diagnosis in a mock-BSS trial in which the victim killed their IPV abuser. Results indicated that jurors were more lenient with female defendants than male defendants. Jurors with less hostile sexism attitudes were more lenient than jurors with who had more hostile sexism attitudes and jurors with more benevolent sexism attitudes were more lenient in their decisions. The PTSD diagnosis did not influence verdict, but jurors found their decisions to be harder when there was a diagnosis. This research provides additional evidence that male and female defendants are not treated equally in the same circumstances despite an increase in egalitarian views. Given that IPV is unfortunately likely to continue, researchers should continue to examine how cases involving BSS are perceived in court. Future research would benefit by examining homosexual relationships, as well as cases involving trans or non-binary individuals. Through the empirical research on this topic, we begin to create increased awareness of IPV risks, particularly in courtroom contexts. We want to promote efforts that result in availability of resources for survivors. We encourage anyone who has experienced IPV to reach out to the National Domestic Hotline at (800) 799-7233 or visit their website at https://www.thehotline.org.

## Supplemental Material

sj-docx-1-jiv-10.1177_08862605241257594 – Supplemental material for The Role of Defendant Gender and PTSD Diagnosis in a Battered Spouse Case Supplemental material, sj-docx-1-jiv-10.1177_08862605241257594 for The Role of Defendant Gender and PTSD Diagnosis in a Battered Spouse Case by Rebekah Adair-Russell, Krystia Reed and Maria F. Torres in Journal of Interpersonal Violence

## Notes

1. For the purposes of this paper, we refer to the concept as BSS, which is the term usually used in the legal system (Sokoloff & Dupont, 2005). However, we recognize anyone in a romantic relationship may experience domestic abuse, regardless of marital status.
