# Supplementary material for: The Role of Defendant Gender and PTSD Diagnosis in a Battered Spouse Case
Source: J Interpers Violence. 2024 Jun 22;40(5-6):1112–34. doi: 10.1177/08862605241257594 (PMC11800720; doi:10.1177/08862605241257594)
Supplement: sj-docx-1-jiv-10.1177_08862605241257594 – Supplemental material for The Role of Defendant Gender and PTSD Diagnosis in a Battered Spouse Case [file sj-docx-1-jiv-10.1177_08862605241257594.docx]

**Table 1**

*Demographic Information*

| Variable | % | *N* |
| --- | --- | --- |
| Gender  Male  Female  Other | 30.1  69.5  0.4 | 71  164  1 |
| Ethnicity  Hispanic  Non-Hispanic | 86.9  12.7 | 205  30 |
| Race  Black or African American  Asian or Pacific Islander  American Indian or Alaskan Native  White  Not Selected | 2.1  2.1  0.4  21.6  73.3 | 5  5  1  51  173 |
| Education  High School Diploma  Some College  College Graduate  Graduate Degree | 14.8  80.5  3.8  0.8 | 35  190  9  2 |
| Political Views  Extremely Liberal  Somewhat Liberal  Lean Liberal  In the Middle  Lean Conservative  Somewhat Conservative  Extremely Conservative | 13.1  19.9  11.0  39.8  10.2  4.7  1.3 | 31  47  26  94  24  11  3 |
| Political Party  Democrat  Republican  Independent  Not political | 39.8  11.4  14.8  33.9 | 94  27  35  80 |

**Table 2**

*Frequencies of Familiarity with IPV*

|  | % | *n* |
| --- | --- | --- |
| Familiarity to IPV – Real-Life  Never  Rarely  Occasionally  Frequently  Very Frequently  *Missing* | 44.9  26.3  16.1  5.9  5.1  1.7 | 106  62  38  14  12  4 |
| Familiarity to IPV – Media  Never  Rarely  Occasionally  Frequently  Very Frequently  *Missing* | 5.5  9.7  37.3  28.4  17.4  1.7 | 13  23  88  67  41  4 |

**Table 3**

*Correlations among Dependent Variables, Independent Variables, and Covariates*

| Variable | *M* | *SD* | 1 | 2 | 3 | 4 | 5 | 6 | 7 | 8 | 9 | 10 | 11 | 12 |
| --- | --- | --- | --- | --- | --- | --- | --- | --- | --- | --- | --- | --- | --- | --- |
| 1 Binary Verdict | .71 | .46 | - |  |  |  |  |  |  |  |  |  |  |  |
| 2 Scaled Verdict | 1.85 | 3.83 | .97** | - |  |  |  |  |  |  |  |  |  |  |
| 3 Difficulty | 1.01 | 3.3 | .23** | .228** | - |  |  |  |  |  |  |  |  |  |
| 4 Defendant Gender | .56 | .497 | -.21** | -.189** | -.028 | - |  |  |  |  |  |  |  |  |
| 5 Defendant PTSD | .49 | .5 | .07 | .048 | .139* | .053 | - |  |  |  |  |  |  |  |
| 6 Participant Gender | .70 | .460 | .04 | .037 | -.030 | -.077 | .112 | - |  |  |  |  |  |  |
| 7 Victim Blaming | 17.32 | 6.24 | -.07 | -.074 | -.092 | -.077 | -.078 | -0.91 | - |  |  |  |  |  |
| 8Hostile Sexism | 1.33 | .848 | .01 | .018 | -.017 | .028 | -.113 | -.255** | .469** | - |  |  |  |  |
| 9 Benevolent Sexism | 2.192 | .817 | -.12 | -.159* | -.033 | -.048 | -.060 | -.195** | .300** | .389** | - |  |  |  |
| 10 Mental Health Stigma | 59.07 | 10.512 | -.06 | -.073 | .050 | -.021 | -.068 | -.051 | .232** | .302** | .226** | - |  |  |
| 11 Familiarity by Contact | .98 | 1.16 | .06 | .066 | .068 | -.126 | -.045 | .193** | .040 | -.125 | .000 | .055 | - |  |
| 12 Familiarity by Media | 2.44 | 1.07 | -.04 | -.021 | .061 | -.094 | .014 | .142* | .036 | -.051 | .062 | .116 | .407** | - |

***p* < .01

**p* < .05

**Table 4**

*ANCOVA of Scaled Verdict on Defendant Gender, Defendant PTSD, and Covariates*

| Predictor | *df* | *F* | Partial $\eta^{2}$ | 95% CI  [LL, UL] |
| --- | --- | --- | --- | --- |
| (Intercept) | 1 | 1.12 | .005 | [-44.89, 70.28] |
| Defendant Gender | 1 | 6.06* | .027 | [9.53, 68.87] |
| Defendant PTSD | 1 | 1.46 | .007 | [-24.16, 34.32] |
| Participant Gender | 1 | .04 | .000 | [-33.98, 39.11] |
| Defendant Gender * Defendant PTSD | 1 | 3.82 | .017 | [-65.10, 20.79] |
| Defendant Gender * Participant Gender | 1 | .08 | .000 | [-50.07, 73.51] |
| Defendant PTSD * Participant Gender | 1 | .37 | .002 | [-45.00, 54.73] |
| Defendant Gender * Defendant PTSD * Participant Gender | 1 | .73 | .003 | [-115.35, 45.54] |
| Victim Blaming | 1 | 1.60 | .007 | [-2.69, .59] |
| Hostile Sexism | 1 | 5.11* | .023 | [1.89, 27.58] |
| Benevolent Sexism | 1 | 6.35* | .029 | [-27.24, -3.33] |
| Mental Health Stigma | 1 | 1.38 | .006 | [-1.45, .37] |
| Familiarity in Contact | 1 | 1.71 | .008 | [-2.87, 14.17] |
| Familiarity in Media | 1 | .11 | .001 | [-10.86, 7.69] |
| Error | 216 |  |  |  |

Note: **p* < .05, ***p* < .01

**Table 5**

*ANCOVA of Difficulty on Defendant Gender, Defendant PTSD, and Covariates*

| Predictor | *df* | *F* | Partial $\eta^{2}$ | 95% CI  [LL, UL] |
| --- | --- | --- | --- | --- |
| (Intercept) | 1 | 15.42 | .067 | [24.27, 73.64] |
| Defendant Gender | 1 | .00 | .000 | [-13.75, 11.69] |
| Defendant PTSD | 1 | 5.75* | .026 | [-23.71, 1.36] |
| Participant Gender | 1 | 1.64 | .008 | [-10.97, 20.36] |
| Defendant Gender * Defendant PTSD | 1 | 1.11 | .005 | [-8.04, 28.78] |
| Defendant Gender * Participant Gender | 1 | .87 | .004 | [-15.12, 37.86] |
| Defendant PTSD * Participant Gender | 1 | 1.01 | .005 | [-10.77, 31.98] |
| Defendant Gender * Defendant PTSD * Participant Gender | 1 | 4.98* | .023 | [-73.54, -4.57] |
| Victim Blaming | 1 | 2.78 | .013 | [-1.30, .11] |
| Hostile Sexism | 1 | .35 | .002 | [-3.85, 7.16] |
| Benevolent Sexism | 1 | .22 | .001 | [-6.33, 3.92] |
| Mental Health Stigma | 1 | .99 | .005 | [-.19, .59] |
| Familiarity in Contact | 1 | 1.60 | .007 | [-1.31, 5.99] |
| Familiarity in Media | 1 | .75 | .003 | [-2.23, 5.72] |
| Error | 216 |  |  |  |

Note: **p* < .05, ***p* < .01
